# Modelling the In Vivo and Ex Vivo DNA Damage Response after Internal Irradiation of Blood from Patients with Thyroid Cancer

**DOI:** 10.3390/ijms25105493

**Published:** 2024-05-17

**Authors:** Sarah Schumann, Harry Scherthan, Philipp E. Hartrampf, Lukas Göring, Andreas K. Buck, Matthias Port, Michael Lassmann, Uta Eberlein

**Affiliations:** 1 Department of Nuclear Medicine, University Hospital Würzburg, 97080 Würzburg, Germany; 2 Bundeswehr Institute of Radiobiology Affiliated to the University of Ulm, 80937 Munich, Germany

**Keywords:** γ-H2AX, 53BP1, radiobiology, modelling, DNA damage, DNA damage repair, dosimetry

## Abstract

This work reports on a model that describes patient-specific absorbed dose-dependent DNA damage response in peripheral blood mononuclear cells of thyroid cancer patients during radioiodine therapy and compares the results with the ex vivo DNA damage response in these patients. Blood samples of 18 patients (nine time points up to 168 h post-administration) were analyzed for radiation-induced γ-H2AX + 53BP1 DNA double-strand break foci (RIF). A linear one-compartment model described the absorbed dose-dependent time course of RIF (Parameters: *c* characterizes DSB damage induction; *k*_1_ and *k*_2_ are rate constants describing fast and slow repair). The rate constants were compared to ex vivo repair rates. A total of 14 patient datasets could be analyzed; *c* ranged from 0.012 to 0.109 mGy^−1^, *k*_2_ from 0 to 0.04 h^−1^. On average, 96% of the damage is repaired quickly with *k*_1_ (range: 0.19–3.03 h^−1^). Two patient subgroups were distinguished by *k*_1_-values (*n* = 6, *k*_1_ > 1.1 h^−1^; *n* = 8, *k*_1_ < 0.6 h^−1^). A weak correlation with patient age was observed. While induction of RIF was similar among ex vivo and in vivo, the respective repair rates failed to correlate. The lack of correlation between in vivo and ex vivo repair rates and the applicability of the model to other therapies will be addressed in further studies.

## 1. Introduction

In order to better understand and further improve the safety and efficacy of radiopharmaceutical therapies, research on the cellular DNA damage response and radiobiology is important [1]. Of particular interest for the safety of a treatment is the investigation of the induction and repair of radiation-induced DNA damage in peripheral blood mononuclear cells (PBMCs), a surrogate for bone marrow sensitivity. In recent years, the γ-H2AX + 53BP1 assay has been widely used to quantify radiation-induced DNA double-strand breaks (DSBs) by counting microscopic foci containing these biomarkers [2,3,4]. Especially for PBMCs, the method is now well-established. Prediction of radiation toxicity and risk assessment of late effects of the treatment may be possible by studying the kinetics of DNA damage in PMBCs [5,6].

Although there is a large body of work after external exposure to ionizing radiation, the number of studies describing the absorbed dose-dependent DNA damage response after internal exposure to radionuclides in PBMCs is limited [7,8,9,10,11,12,13,14,15]. The fundamental difference between radiopharmaceutical therapies and external irradiation of patients is that the activity in the body and blood of patients is present and can be measured over a period of weeks, which leads to persistent induction of DNA damage by the residual activity that competes with ongoing repair. Furthermore, absorbed doses to the blood after therapeutic exposure to β-emitters are in the range of hundreds of milligray [7,10,11,12].

To date, only two of the published patient studies investigating DSB damage in PBMCs after internal irradiation have attempted to quantify the absorbed dose-dependent time course of DNA DSBs by using a simplified empirical mathematical model [10,11]. The challenge for the development of a robust model is that there is a lack of suitable datasets of patients with a sufficient number of uniformly sampled data points in combination with dosimetry. This was also a shortcoming of our previous study on patients after radioiodine therapy, as, at that time, the patient sampling time points were focused on the DNA damage induction shortly after administration of [^131^I]NaI, and not on late time points which govern the repair of DNA damage [11].

Therefore, the aim of this investigation was, in a prospective patient study, to collect more uniform data on radiation-induced γ-H2AX and 53BP1 DSB foci in combination with blood dosimetry at sufficient and well-defined early and late time points. The objective was to generate patient-specific datasets of γ-H2AX + 53BP1 foci for developing a generally valid model that describes the induction and repair of DNA DSBs in blood cells during radioiodine therapy. These data were collected and evaluated from patients with differentiated thyroid carcinoma during their first radioiodine therapy as part of a sub-study of the EU-funded Horizon 2020 MEDIRAD project [16,17,18]. The primary aim was to obtain information on the variability of the in vivo DSB damage induction and repair in this patient group and to link it to clinical observations. Furthermore, the mathematical model implemented in this work should be used to establish a comparison between in vivo and ex vivo DSB induction and repair. It is of note that the DSB induction and repair has been previously investigated ex vivo in the same patient cohort [17] to compare whether an ex vivo irradiation with similar absorbed doses and dose rates allows for a prediction of the in vivo repair rates.

## 2. Results

### 2.1. Patient Data

Eighteen patients (fourteen female and four male) aged between 19 and 65 years were included in the study. For their first radioiodine therapy, they received a median activity of 3.57 GBq [^131^I]NaI (min: 3.43 GBq, max: 3.91 GBq). Their demographic and clinical data are listed in Table 1. One patient (IP15) had recombinant human thyroid-stimulating hormone (rhTSH) administered before therapy start, while all other patients were treated under thyroid hormone withdrawal (THW). Analysis of the follow-up data revealed that four patients (IP4, IP6, IP9, and IP15) received a further radioiodine therapy.

There were occasional deviations from the nominal time points for blood sampling due to clinical routines, and in some cases, blood samples could not be drawn or analyzed. Data for the nominal time point at 3 h after administration are missing for patient IP3. Data for the nominal time point at 96 h after administration are missing for patients IP6, IP8, IP9, IP10, IP11, and IP12. Data for the nominal time point at 168 h after administration are missing for patient IP10 and were collected one day earlier for patient IP15.

### 2.2. Absorbed Doses to the Blood and Number of RIF

Absorbed doses to the blood were calculated for all of the nominal time points investigated. The median values for these time points are shown in Table 2. For patient IP15, we had to adjust our absorbed dose to the blood calculation routine. Unlike the other patients, for this patient, a new increase in the activity in the blood was observed 96 h after administration. Therefore, *τ_bl_* was fitted bi-exponentially only up to the time point 48 h after administration and then completed section by section.

**Figure 1:**
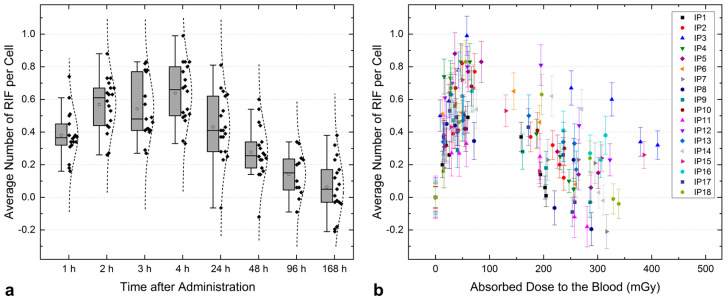
(**a**) Average number of RIF per cell at the eight nominal time points after [^131^I]NaI administration shown as a boxplot including the data points of the 18 patients (black diamonds) with a fit of normal distribution (black dotted line). (**b**) Average number of RIF per cell at the consecutive time points as a function of the absorbed dose to the blood.

The median total absorbed dose to the blood of these patients is 299 mGy (min: 205 mGy, max: 605 mGy) in agreement with those determined in previous studies (see, e.g., [7,11,19]). Absorbed doses to the blood of more than 1 Gy after ablation treatment with 3.7 GBq have been reported. The administration of higher activities than 3.7 GBq would still result in absorbed doses to the blood below 2 Gy for most patients, a value up to which Rothkamm and Löbrich [20] observed a linear relationship for γ-H2AX foci formation. The median values of the average number of RIF per cell at each time point are reported in Table 2. In most patients (16/18), the maximum number of RIF per cell is reached within the first 4 h after administration. Thereafter, a decrease could be observed. After 168 h, the average number of RIF per cell reached zero in seven out of seventeen patients. The average number of RIF per cell *N* at the eight nominal time points is shown in Figure 1a.

**Figure 2:**
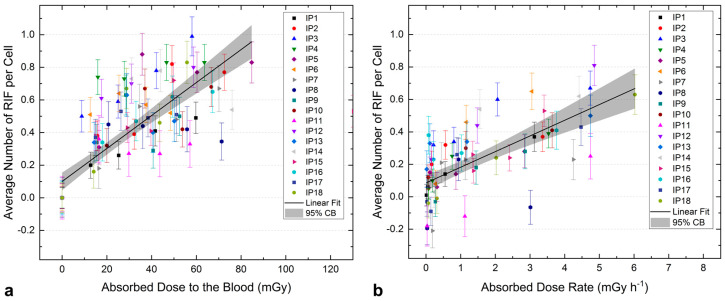
Average number of RIF per cell as a function of the absorbed dose to the blood (**a**) and the absorbed dose rate (**b**). (**a**) Absorbed dose dependence: detailed view of the first five data points corresponding to the blood samples taken at the day of [^131^I]NaI administration, including a linear fit to the pooled data with 95% confidence band. (**b**) Absorbed dose rate dependence: detailed view of the last four data points (time points ≥ 24 h after [^131^I]NaI administration), including a linear fit to the pooled data with 95% confidence band.

The average number of RIF per cell *N* as a function of the absorbed dose to the blood *D* is shown in Figure 1b. To additionally compare the induction of RIF as a function of the absorbed dose to a previous study [11] and check for linearity during the first hours after the start of therapy, a simple linear fit of the pooled patient data was performed considering the first five data points (Figure 2a). This resulted in the following equation for the absorbed dose dependence of the average number of RIF per cell (*r^2^* = 0.64):

(1) $$N\left(D\right)=\left(0.010\pm 0.001\right)mGy^{-1}\cdot D+\left(0.100\pm 0.027\right)\;for\;t<6\;h$$

Also, at later time points, patients with high absorbed doses to the blood (such as IP3 and IP15) usually show a higher number of RIF per cell compared to other patients (see Figure 1b). In general, the change in the number of RIF per cell over time is characterized by an increasing absorbed dose with decreasing dose rate. A dependence between the average number of RIF per cell at time points > 24 h after administration and the dose rate was shown in a previous study in prostate cancer patients during therapy with [^177^Lu]Lu-PSMA [12] and also tested here, including all data points for *t* ≥ 20 h. Figure 2b shows the average number of RIF per cell as a function of the absorbed dose rate, including a linear fit of the pooled data with the following functional equation (*r^2^* = 0.49):

(2) $$N\left(\frac{dD}{dt}\right)=\left(0.097\pm 0.012\right)\;h\;mGy^{-1}\cdot \frac{dD}{dt}+\left(0.085\pm 0.024\right)\;for\;t\geq 20\;h$$

### 2.3. Modelling

The number of RIF as a function of time after administration was fitted according to Equation (4) for all of the 18 patients individually, taking into account the obtained patient-specific blood dosimetry data. For patient IP15, to ensure comparability between all patients, dosimetry data from a bi-exponential fit over the entire range were used for the modelling. In total, four patients had to be excluded due to missing data points (IP10 and IP12 with only seven data points) or large errors of the fit parameters (IP8 and IP11 with errors greater than twice the value of the parameter). In all other patients, data are described well by the model, with coefficients of determination *r^2^* ≥ 0.81.

For testing the robustness of the fitting, we repeated the fitting procedures by varying the parameters and by reducing the number of data points to 8. The variation in the fitting parameters was in the order of 10% or less. For determining *k*_2_, the last time point at *t* = 196 h determines its value; hence, this time point was crucial. We also consider the early time points (up to 4 h after administration) to be crucial to measure the induction of RIF, as was shown in previous publications [10,11,12]. Additionally, a simplified fit considering only one repair rate *k*_1_ (*α* = 1) provided correlation coefficients (except for one patient, who only showed a fast repair component) that were considerably lower than those obtained from the more sophisticated model.

On average, 96% of the damage is repaired with *k*_1_ varying between 0.19 h^−1^ and 3.03 h^−1^, while the rest is repaired with *k*_2_ ranging from 0 to 0.04 h^−1^. The induction constant *c* is 0.026 mGy^−1^ at the median (min: 0.012, max: 0.109). The mean value for parameter *N_0_* is −0.018 ± 0.032. The resulting fit parameters of all individual patient data fits are listed in Table 3.

**Figure 3:**
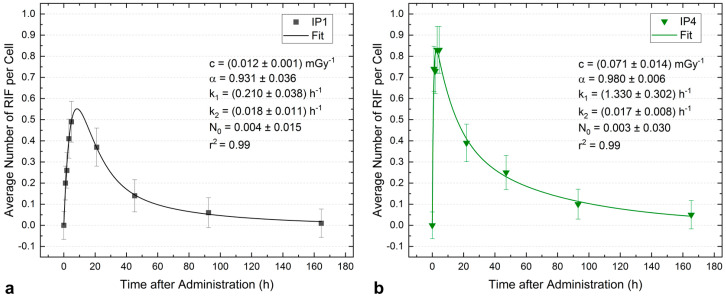
Modelled time course of the average number of RIF per cell exemplified by two selected patients. (**a**) Patient IP1 who is assigned to Group 1. (**b**) Patient IP4 who is assigned to Group 2.

In particular, the values for *k*_1_ vary strongly among patients and are either below 0.6 h^−1^ or above 1.1 h^−1^, which allowed the assortment of patients into two groups: patients with slower repair kinetics (Group 1, *n* = 8, *k*_1_ < 0.6 h^−1^) and patients with faster repair kinetics (Group 2, *n* = 6, *k*_1_ > 1.1 h^−1^). To illustrate the differences, the average number of RIF per cell as a function of time after administration and the respective absorbed dose-dependent fit functions are shown in Figure 3a for one patient (IP1) assigned to Group 1 and in Figure 3b for one patient (IP4) assigned to Group 2. In addition, the values of the variable fit parameters are shown. Additionally, we performed a k-means clustering analysis for the variables *k*_1_, *k*_2_, and *c* (Supplementary Figure S1). For this analysis, patient IP6 was identified as an outlier. For the remaining 12 patients with a complete dataset of fit parameters *k*_1_, *k*_2_, and *c*, the results (Group 1: *k*_1_ = 0.37429 h^−1^, *c* = 0.02143 mGy^−1^, *k*_2_ = 0.00657 h^−1^, Group 2: *k*_1_ = 1.3208 h^−1^, *c* = 0.052 mGy^−1^, *k*_2_ = 0.0276 h^−1^; *p* < 0.0001 for *k*_1_ and *c*; *p* = 0.012 for *k*_2_) are in good agreement with the values obtained by the pooled fitting of the two patient groups. This further validates our approach of grouping the patient data.

**Figure 4:**
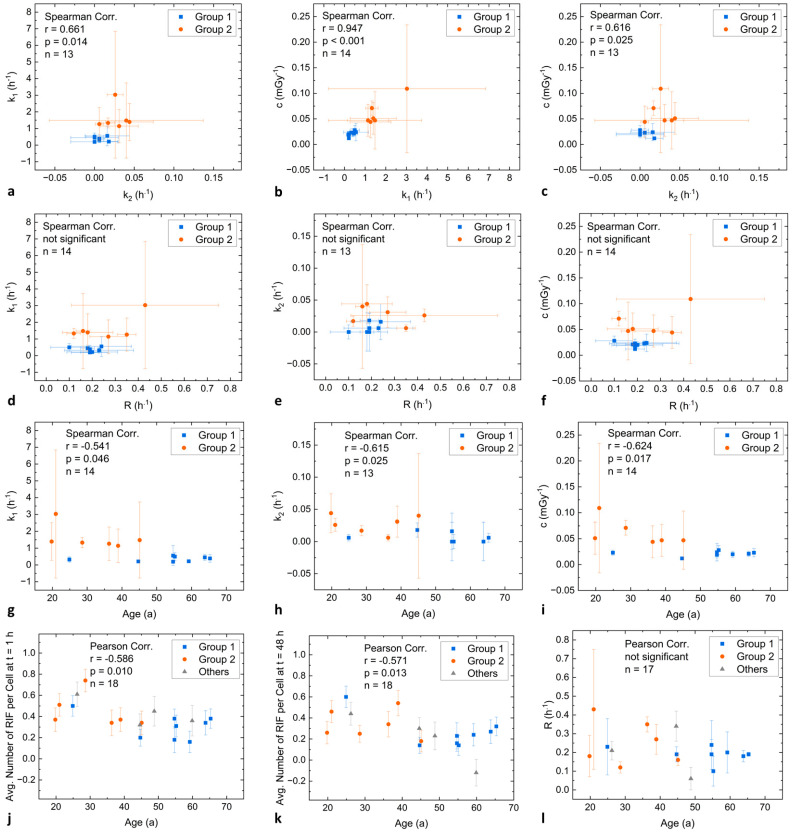
Correlation analysis. Data of patients assigned to Group 1 are shown in blue, while data of patients from Group 2 are shown in orange. Data of patients excluded from the modelling analyses are shown in grey (Others). (**a**–**c**): Correlation and corresponding correlation coefficients of the fit parameters *k*_1_, *k*_2_, and *c*. (**d**–**f**): Correlation of the fit parameters with the ex vivo repair rate *R*. (**g**–**k**): Correlation and corresponding correlation coefficients of the fit parameters and the average number of RIF per cell 1 h and 48 h after administration with the age of the patients. (**l**): Correlation and corresponding correlation coefficients of the ex vivo repair rate *R* with the age of the patients.

*k*_1_-values correlate significantly with *k*_2_-values (Spearman correlation, *r* = 0.661, *p* = 0.014; Figure 4a) and *k*_2_-values are significantly different between Group 1 and Group 2 (Mann-Whitney U test, *p* = 0.017). There is also a significant correlation between *k*_1_ and the damage induction rate *c* (Spearman correlation, *r* = 0.947, *p* < 0.001; Figure 4b) and *k*_2_ and *c* (Spearman correlation, *r* = 0.616, *p* = 0.025; Figure 4c) for all patients. Accordingly, also the values for *c* are significantly different between Group 1 and Group 2 (Mann-Whitney U test, *p* = 0.002). In contrast, there is no significant difference between the two groups for the parameter *α* that denotes the proportion of damage repaired with *k*_1_.

**Figure 5:**
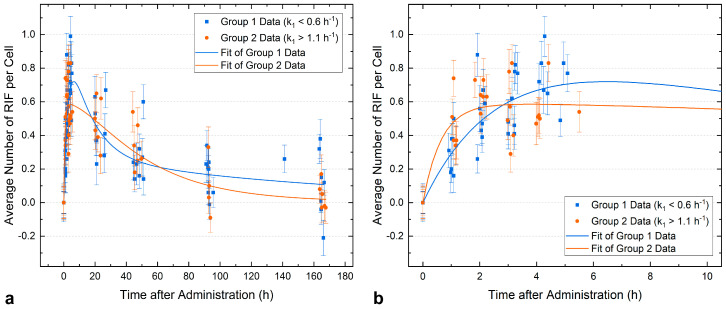
Average number of RIF per cell as a function of the time after administration (*n* = 14). The resulting fits according to Equation (4) are shown for the pooled data of Group 1 (blue, *n* = 8) and the pooled data of Group 2 (orange, *n* = 6). (**a**) Overview of all data points. (**b**) Detailed view of the first five data points corresponding to the blood samples taken at the day of [^131^I]NaI administration.

Due to the marked differences in repair kinetics among patients, we refrained from performing a pooled fit of all patient data. Instead, data from Group 1 and Group 2 were pooled separately and two independent fits were performed. The results for each of the parameter sets are shown in Figure 5 and in Table 4. Figure 5 gives an overview of all data points (Figure 5a) and a detailed view of the first five data points corresponding to the blood samples taken at the day of [^131^I]NaI administration to better visualize the initial phase of DSB induction (Figure 5b). According to the resulting fit functions, a steeper increase in RIF per cell in the first 2 h is observed for Group 2 compared to Group 1, as shown in Figure 5b. The maximum number of RIF per cell is reached after 6.5 h for Group 1 and after 3.7 h for Group 2 and amounts to 0.72 and 0.59, respectively. However, the course of DNA repair, as indicated by the decrease in RIF per cell with time, is faster for Group 1. At the last time point investigated, 168 h after [^131^I]NaI administration, 0.11 RIF per cell can be calculated for Group 1 and 0.02 RIF per cell for Group 2.

### 2.4. Comparison with Ex Vivo Data

Further analyses examined correlations between the in vivo repair rates *k*_1_ and *k*_2_ and the repair rate *R* determined after ex vivo irradiation of the same patients’ blood (*n* = 14). The ex vivo data for these analyses were taken from a previous publication [17] and are listed in Table 3. The reduction from initially 33 patients in the ex vivo study to 18 patients allowed for a patient-by-patient comparison of blood samples irradiated in vivo and ex vivo because only 18 had matching datasets. Therefore, we included patient data of only those patients and compared these data pairwise. The intention was to test if the ex vivo irradiation with similar absorbed doses and dose rates allows for a prediction of the in vivo repair rates.

No significant correlations were found, neither between *k*_1_ and *R* nor between *k_2_* and *R*, and *c* and *R* (Figure 4d–f). However, it is remarkable that the patient with the highest repair rate *k*_1_ in vivo (IP6) also had the highest repair rate *R* after ex vivo irradiation of his blood, compared to the other patients (Figure 4d).

**Figure 6:**
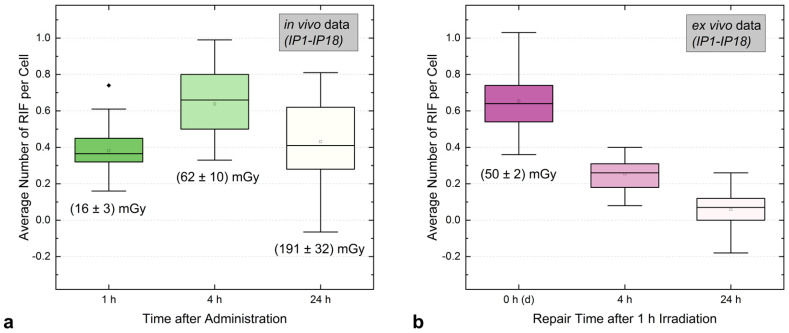
Boxplot of the in vivo (**a**) and ex vivo (**b**) RIF induction and repair over the first 24 h after [^131^I]NaI administration (in vivo) or irradiation with [^131^I]NaI (ex vivo) resulting in an absorbed dose to the blood of 50 mGy after 1 h incubation. In panel (**a**), the mean absorbed doses to the blood at the nominal time points after administration are shown. In panel (**b**), the repair time after the completed ex vivo irradiation is represented on the X-axis. Consequently, the first box shows the average number of RIF per cell induced without additional repair time (0 h), i.e., directly (d) after 1 h of internal irradiation.

Figure 6 demonstrates the differences between the temporal pattern of DNA damage induction and repair between this study and the ex vivo study for three time points after administration (in vivo) or after 1 h irradiation with [^131^I]NaI (ex vivo) for the same 18 patients IP1-IP18 [17,18]. In vivo, the mean absorbed dose to the blood was (16 ± 3) mGy 1 h after administration, (62 ± 10) mGy 4 h after administration, and (191 ± 32) mGy 24 h after administration. Ex vivo, the absorbed dose to the blood was (50 ± 2) mGy after 1 h irradiation, followed by removal of radioactivity and direct cell fixation (0 h (d)), and after 4 h and 24 h repair time. For these three time points, the mean of the average number of RIF per cell was 0.38 ± 0.14, 0.64 ± 0.18, and 0.43 ± 0.20 for the in vivo study and, for the ex vivo study, 0.65 ± 0.18, 0.25 ± 0.09, 0.06 ± 0.09.

For similar absorbed doses (ex vivo: (50 ± 2) mGy, in vivo: (62 ± 10) mGy), comparable mean values of the average numbers of RIF per cell (ex vivo: 0.65 ± 0.18, in vivo: 0.64 ± 0.18) were obtained, indicating that the induction of RIF is similar ex vivo and in vivo.

### 2.5. Correlations of Fit Parameters with Clinical Data

Significant correlations were observed between the patients’ age and both repair rates *k*_1_ (Spearman correlation, *r* = −0.541, *p* = 0.046; Figure 4g) and *k*_2_ (Spearman correlation, *r* = −0.615, *p* = 0.025; Figure 4h) as well as parameter *c* (Spearman correlation, *r* = −0.624, *p* = 0.017; Figure 4i), indicating that repair efficacy decreases with age. The patients’ age also correlated significantly with the average number of RIF 1 h after administration (Pearson correlation, *r* = −0.586, *p* = 0.010; Figure 4j), suggesting an age dependence of the initial RIF induction. Furthermore, the patients’ age correlated with the average number of RIF 48 h after administration (Pearson correlation, *r* = −0.571, *p* = 0.013; Figure 4k), which matches the finding that in vivo repair rates resulting from the modelling correlate significantly with age. In contrast, there was no significant correlation between the ex vivo repair rate and the age of the patients (Figure 4l).

Other clinical parameters obtained throughout the study (patient weight, TSH, fT3, fT4, Tg, creatinine, platelets, white blood cells) did not correlate with *c*, *k*_1_, and *k*_2_.

## 3. Discussion

Data on γ-H2AX + 53BP1 foci in PBMCs after internal irradiation in combination with blood-based dosimetry allowed the development of a mathematical model describing the time-dependent progression of radiation-induced DSB damage during radioiodine therapy for individual patients. This approach allowed for the first time a direct comparison between the resulting DSB repair rates in vivo and ex vivo in blood samples from the same patients.

For the induction of RIF during the first hours after administration, a linear fit resulted in a value of (0.010 ± 0.001) mGy^−1^. This value agrees well with the slope of (0.012 ± 0.001) mGy^−1^ of an in vivo calibration curve implemented for the first two hours after radioiodine administration in a previous study by Eberlein et al. [11]. The fact that this value is significantly lower than the induction constant *c* (0.026 mGy^−1^ in median) determined via the modelling may indicate that even in the first hours after administration, DNA repair makes a substantial, non-negligible contribution. This is consistent with classical non-homologous end-joining (cNHEJ) DNA repair making a major contribution to the fast repair of simple euchromatic DSBs [21,22]. We assumed that the decrease in DSBs was due to DNA repair progression and not caused by apoptotic cells that were removed from the system, since the absorbed doses to the blood were low and the damages seeded in the blood cells were below toxic doses leading to cell death. Only the latter will be cleared from the system, e.g., in the spleen. Apoptotic cells have only been observed in larger quantities in organisms exposed to large acute doses above 0.5 Gy [23,24].

A comparison between in vivo and ex vivo data of the same patients showed that the induction of RIF is similar in both cases at a comparable absorbed dose to the blood. While the absorbed dose determines the number of RIF during the first hours after the start of therapy, the absorbed dose rate is informative for time points ≥ 20 h after administration, as we observed here, in agreement with a previous study [12].

When interpreting the results of the modelling, one of the main findings is that the fit parameters varied substantially between individual patients. This finding prevented a combined fit of the pooled data to provide a generally valid description of the time- and dose-dependent DNA damage progression. Hence, the individual fits were analyzed separately. It appeared that patients could be divided into two groups based on their different repair rates. The categorization in two patient groups with fast repair or slow repair (according to their *k*_1_-values) was considered most appropriate after assessment of the fit parameters determined. In the future, it will be important to check this observation in a larger patient cohort to test whether there will be even more groups to derive a more accurate description, and whether the *k*_1_- and *k*_2_-values derived from our model are predictive for patient outcome or unwanted side effects. This will require more patient studies with different radiopharmaceuticals and diseases.

The choice of the fit function used here for modelling the time course of the number of RIF was based on the aforementioned differential equation assuming an absorbed dose dependence. The intention was to keep the model as simple and general as possible. Therefore, only two different time-constant repair rates were considered, and the uptake of radioiodine from the stomach into the blood during the first 20–30 min after oral administration was not considered separately.

It is a property of the proposed compartment model, which links dose rate, RIF induction, and DSB repair, that the parameters *k*_1_, *k*_2_, and *c* are not independent but correlate. As the induction rate *c* cannot be derived directly and separately from ex vivo experiments, leaving one of the values fixed does not provide meaningful results. For the model proposed in this work, we had to tolerate this bias by the dependencies of the fitting parameters.

A previous study applied a simpler, empirical model to describe the progression of γ-H2AX + 53BP1 DSB foci during radioiodine therapy [11]. That work also considered a fast repair constant, with a value of 0.293 h^−1^, and a slow repair constant, with a value of 0.036 h^−1^. Higher values than 0.66 h^−1^ for the fast repair rate, as determined in this study for Group 2, however, were not observed. According to the model of Eberlein et al. [11], reaching the maximum number of foci is assumed to occur earlier, after 3.2 h, compared to 6.5 h for Group 1 and 3.7 h for Group 2 in the present work. Reasons for these differences could be that, in contrast to the current study, assumptions from earlier ex vivo studies were also considered in the previous model, and that RIF data for the fitting procedure were much less homogeneous. For example, the last data point was 48 h after administration in some patients and there was only one patient with data up to 168 h after administration [11].

A further study investigated DSB repair after internal ex vivo irradiation with [^131^I]NaI in the same patient population, but expanded by 15 patients [17]. The ex vivo repair rate in this case was determined by a mono-exponential fit of the pooled data and was (0.28 ± 0.03) h^−1^, comparable to the repair rate *k*_1_ of Group 1 of (0.36 ± 0.11) h^−1^. Additionally, a (6 ± 2)% proportion of residual damage determined in the ex vivo model fits the finding of the present study that (96 ± 2)% of damage is repaired at a fast repair rate, while the remaining damage is repaired slowly, or not at all [17]. However, significant correlations between the ex vivo repair rate *R* and the in vivo repair rates *k*_1_ and *k*_2_ of the individual patients could not be found, suggesting that the individual in vivo DSB repair efficiency cannot be predicted by less effortful ex vivo analyses. This finding, however, needs to be confirmed in studies with more patients and other irradiation scenarios.

The lack of correlation of in vivo and ex vivo DNA repair rates can be explained by the influence of some factors in vivo which are not present in the ex vivo experiments. The ex vivo setup is a simplified system, since it does not contain serum proteins, has a lower cell density and no systemic circulation and further components. Additionally, ex vivo cell culture conditions generally lack the antioxidant capacity that is present in patients in vivo [25] which would reduce the radicals induced by low-LET systemic irradiation (e.g., [26,27]). Furthermore, the three time points per patient in the ex vivo scenario restricted us to a mono-exponential fit function to determine the ex vivo repair rate. The ex vivo irradiation set-up may be improved; however, our data provide observations that deserve further consideration, e.g., by increasing the number of repair time points and patients.

As our Group 1 contains mostly older individuals (except one; Figure 4g–l), our findings suggest that a faster repair rate is correlated with younger age, while the maximum RIF obtained by the modelling is lower in this group. Since fast NHEJ repair removes simple DSBs soon after their induction [28,29] and given the observation that NHEJ is less efficient in older individuals [30,31], this may explain the higher maximum of the fitted RIF values in this group.

Significant correlations were found between patient age and the in vivo fit parameters *k*_1_, *k*_2_, and *c*, also suggesting that the efficiency of DSB repair decreases with increasing age. This finding is well in line with the results in the literature (e.g., [32,33]). In contrast, no such dependency was observed in the corresponding ex vivo study [17], underlining the importance of in vivo studies. This observation can only be transferred to a limited extent to other similar patient studies during radiopharmaceutical therapies [10,12], as the patients are older on average and their age range is narrow (mean age of patients receiving [^177^Lu]Lu-DOTATATE/DOTATOC in [10]: 61 ± 11 years; mean age of patients receiving [^177^Lu]Lu-PSMA in [12]: 70 ± 9 years).

A recent review by Cheong and Nagel on human variation in DNA repair, immune function, and cancer risk provides more evidence that the DNA damage repair capacity in humans is decreasing with age [33]. This is in line with our observation that faster repair rates are found in younger patients (Group 2) as compared to older patients (Group 1), which is reflected by an age dependency of the repair rates *k*_1_ and *k*_2_ (Figure 4g,h).

Correlations of γ-H2AX and 53BP1 DSB foci induction and repair with clinical parameters were investigated by Derlin et al. [14] in [^177^Lu]Lu-DOTATATE treated patients. While they did not test correlations with the age of the patients, these authors revealed significant correlations between DSB marker kinetics with other clinical parameters, such as a change in platelet count [14], which were not observed in the present study.

As a limitation of our study, it is of note that the validity of the correlation analyses performed here is restricted by the relatively small number of patients as well as the absence of normal distribution of some parameters. In addition, internal, model-based dependencies of the fit parameters may have influenced the results of our correlation analyses.

Our current study focused on the development of a model for DSB damage in PBMCs, since blood samples can be easily obtained. In patients, blood can serve as a surrogate tissue for determining the radiation sensitivity in organs- or tissues-at-risk, such as the bone marrow. This model also has the potential to become an important part of radiation accident preparedness and management after internal irradiation [34,35]. In future, it will be important to determine whether the results of this study can be transferred to other treatment modalities in nuclear medicine and further cell and tissue types.

## 4. Materials and Methods

### 4.1. Patients

A total of 18 patients (IP1-IP18) with differentiated thyroid cancer (DTC) who received their first radioiodine therapy were included in this sub-study within the EU-funded Horizon 2020 MEDIRAD project. The main inclusion criteria have been published previously [16,17] and are provided in the supplement.

Blood samples were collected before and at up to eight different time points after the start of therapy, i.e., 1 h, 2 h, 3 h, 4 h, 24 h, 48 h, 96 h, and 168 h after oral administration of 3.7 GBq [^131^I]NaI, using Li-Heparin blood collecting tubes (S-Monovette; Sarstedt, Nümbrecht, Germany). One part of each blood sample was used for activity quantification and therefore measured in a calibrated high-purity germanium detector (Canberra, Rüsselsheim, Germany). The other part of each blood sample was used to assess DSB damage applying the γ-H2AX + 53BP1 focus assay.

To determine the whole-body activity retention, gamma camera scans were performed at up to six time points after the start of therapy, i.e., 4 h, 24 h, 48 h, 72 h, 96 h, and 168 h after radioiodine administration. Additionally, multiple dose rate measurements were performed using a ceiling-mounted shielded survey meter (automess GmbH, Ladenburg, Germany). The evaluation and normalization of the measurements was carried out as described previously [12].

For correlation analyses, a full set of pretherapeutic values for the following clinical parameters were available: patient age, patient weight, thyroid-stimulating hormone (TSH), free triiodothyronine (fT3), free levothyroxine (fT4), thyroglobulin (Tg), creatinine, platelets, and white blood cells.

### 4.2. Dosimetry

Blood-based dosimetry was performed as described in detail by Eberlein et al. [11]. The results of the measurements of the activity in blood samples for each patient and each blood sample individually and the whole-body activity retention were used to create time–activity curves for the blood and the whole body that were subsequently fitted by bi-exponential functions in order to determine the time-integrated activity coefficients (TIACs) *τ_bl_* for the blood and *τ_wb_* for the whole body. The resulting absorbed dose rate is an intrinsic part of determining individual fitting parameters for each patient. The absorbed dose rate is described by the following function:

(3) $$\frac{dD\left(t\right)}{dt}=A_{0}\cdot \left({Dk}_{\beta }\cdot \frac{{d\tau }_{bl}\left(t\right)}{dt}+{Dk}_{\gamma }\;wt^{-2/3}\cdot \frac{{d\tau }_{wb}\left(t\right)}{dt}\right)$$

Here, *Dk_β_* and *Dk_γ_* are the absorbed dose coefficients for the dose contribution due to self-irradiation of the blood and the dose contribution due to irradiation of the blood by the whole body, respectively. The administered activity is given by *A*_0_ and *wt* denotes the body weight of the patient. A detailed description of the method to derive the absorbed dose to the blood is given by the 2008 “EANM Dosimetry Committee series on standard operational procedures for pre-therapeutic dosimetry I: blood and bone marrow dosimetry in differentiated thyroid cancer therapy” [36].

### 4.3. Evaluation of DSB Damage

To assess DNA DSB damage, co-localizing γ-H2AX + 53BP1 stained foci in isolated and ethanol-fixed peripheral blood mononuclear cells (PBMCs) were quantified blinded by an experienced operator (HS) in 100 cells per sample by manual inspection using a Zeiss Axioimager Z2 epifluorescence microscope equipped with green/red double-band-pass filters (Chroma technology, Olching, Germany). Only the co-localizing foci were counted to exclude artifacts, as γ-H2AX is also formed upon exposure to other inducing agents, e.g., toxins, heat, etc. [37], while 53BP1 is a damage-associated protein occurring early after DSB formation [38] that promotes NHEJ repair [39]. 53BP1 and γ-H2AX foci have been found to be reliable DSB markers in biodosimetry up to 24 h after low-dose low-LET irradiation at absorbed doses below 2 Gy [40,41,42]. The co-localization of both markers ensures that DSB-related foci are addressed, avoiding artefacts in the repair model, as γ-H2AX foci persisting beyond DSB repair [43] are excluded. The PBMC isolation and fixation as well as the immunofluorescent staining process and subsequent evaluation of DSB foci followed a protocol that is described in detail in a previous publication [17].

The number of radiation-induced γ-H2AX + 53BP1 foci (RIF) in each sample was calculated by subtracting the number of patient-specific baseline foci, i.e., the number of foci counted in the corresponding non-irradiated sample that was taken before the start of therapy. For a subset of irradiated and background samples, we replicated the same measurements on the same blood sample 3 times. The variability of the number of foci was within the counting uncertainty. For the uncertainty of focus counting, we assumed a Poisson distribution and, for the uncertainty of the RIF values, we also included error propagation.

### 4.4. Modelling the DNA Damage Response

For modelling the time course of the number of RIF, *N*(*t*), a linear one-compartment model has been developed. The time-dependent induction of RIF is assumed to be proportional to the dose rate d*D*(*t*)/d*t* (proportionality factor *c*, unit: mGy^−1^). The decrease in *N*(*t*) by DNA damage repair is proportional to the number of RIF, with a proportionality factor characterized by a time-constant repair rate *k* (unit: h^−1^). This results in the following differential equation:

(4) $$\frac{dN\left(t\right)}{dt}=-k\cdot N\left(t\right)+c\cdot \frac{dD\left(t\right)}{dt}$$

Simple DSBs are assumed to be repaired with faster kinetics than complex DSBs or DSBs in dense chromatin regions [44,45], which highlights that not all DNA damage repair can be described by a single constant repair rate *k*. In accordance with existing models [10,11,22,46,47], it was therefore assumed that a proportion *α* of RIF is repaired faster (with rate *k*_1_) than the rest (with rate *k*_2_):

(5) $$\begin{array}{cc} \frac{dN\left(t\right)}{dt} & =\frac{dN_{1}\left(t\right)}{dt}+\frac{dN_{2}\left(t\right)}{dt}\;\\ & =\alpha \cdot \left(-k_{1}\cdot N_{1}\left(t\right)+c\cdot \frac{dD\left(t\right)}{dt}\right)+\left(1-\alpha \right)\cdot \left(-k_{2}\cdot N_{2}\left(t\right)+c\cdot \frac{dD\left(t\right)}{dt}\right) \end{array}$$

Integrating the differential equation results in the following function:

(6) $$\begin{array}{cc} N\left(t\right)=c\cdot A_{0}\cdot & ((Dk_{\beta }\sum_{i=1}^{2}\frac{a_{i,bl}}{k_{2}-{(l}_{i,bl}+l_{phys})}\cdot (\exp\left(-{(l}_{i,bl}+l_{phys})\;t\right)-\exp\left(-k_{2}t\right))\\ & +Dk_{\gamma }\;wt^{-\frac{2}{3}}\sum_{i=1}^{2}\frac{a_{i,wb}}{k_{2}-{(l}_{i,wb}+l_{phys})\;}\cdot (\exp\left(-{(l}_{i,wb}+l_{phys})\;t\right)-\exp\left(-k_{2}t\right)))\\ & +\alpha (Dk_{\beta }\sum_{i=1}^{2}\frac{a_{i,bl}}{k_{1}-{(l}_{i,bl}+l_{phys})}\cdot (\exp\left(-{(l}_{i,bl}+l_{phys})\;t\right)-\exp\left(-k_{1}t\right))\\ & +Dk_{\gamma }\;wt^{-\frac{2}{3}}\sum_{i=1}^{2}\frac{a_{i,wb}}{k_{1}-{(l}_{i,wb}+l_{phys})\;}\cdot (\exp\left(-{(l}_{i,wb}+l_{phys})\;t\right)-\exp\left(-k_{1}t\right)))\\ & +\left(1-\alpha \right)(Dk_{\beta }\sum_{i=1}^{2}\frac{a_{i,bl}}{k_{2}-{(l}_{i,bl}+l_{phys})}\cdot (\exp\left(-{(l}_{i,bl}+l_{phys})\;t\right)-\exp\left(-k_{2}t\right))\\ & +Dk_{\gamma }\;wt^{-\frac{2}{3}}\sum_{i=1}^{2}\frac{a_{i,wb}}{k_{2}-{(l}_{i,wb}+l_{phys})\;}\cdot (\exp\left(-{(l}_{i,wb}+l_{phys})\;t\right)-\exp\left(-k_{2}t\right))))\\ & +N_{0} \end{array}$$

The parameters *a_i,bl_* and *l_i,bl_* as well as *a_i,wb_* and *l_i,wb_* correspond to patient-specific blood dosimetry data which resulted from bi-exponential fits of the time–activity curves for the blood (index *bl*) and the whole body (index *wb*). *l_phys_* is the decay constant for the pure physical decay of ^131^I (0.00360 h^−1^). To account for the uncertainty of the number of RIF at time *t* = 0, the intercept *N*_0_ was implemented.

### 4.5. Fitting Process

For the fit of *N*_0_, bounds were set such that the parameter must be within the uncertainty of the experimental data. For *k*_1_ and *k*_2_, all values > 0 were allowed, while *α* was restricted to values between 0 and 1. The function was implemented in OriginPro 2023 (OriginLab Corporation, Northampton, MA, USA). Each patient dataset, weighted with the respective uncertainties, was fitted independently to determine the patient-specific absorbed dose-dependent time course of the number of RIF. For calculation of the five adjustable parameters (*c*, *α*, *k*_1_, *k*_2_, *N*_0_), the Levenberg-Marquardt algorithm was applied until a χ^2^ tolerance value of 10^−9^ was reached. Fitting was performed if eight or more valid data points were available.

### 4.6. Statistical Analysis

Statistical data analysis and plotting was performed with the software OriginPro 2023 (OriginLab Corporation, Northampton, MA, USA). Data were tested for normal distribution using the Shapiro-Wilk test. For correlation analyses, Pearson’s method was applied when normal distribution was confirmed and Spearman’s method was applied when normal distribution was rejected. For testing for differences, *t*-tests were chosen accordingly for normally distributed datasets and Mann-Whitney U tests for non-normally distributed datasets. For all statistical tests, the significance level was set at 5%.

## 5. Conclusions

This study developed a model that is well-suited to describe patient-specific induction of DSB damage and its repair in PBMCs during radioiodine therapy. The pronounced differences in the fit parameters show that induction and repair rates vary significantly between patients and cannot easily be summarized as standard parameters for one “model patient”. It is thus reasonable to consider individual patients separately or group patients with similar features of DSB induction and repair. Our model is not restricted to radioiodine therapies but can easily be extended to other treatments with radiopharmaceuticals to provide further insights whether and how treatment schemes can be adapted to individual patients, e.g., for consecutive cycles in the treatment of prostate cancer with radioligands. However, the lack of a significant correlation between DNA damage repair in ex vivo and in vivo studies, and a potential age dependency of DNA damage repair need to be addressed in further studies. Overall, the findings underline the relevance of biodosimetry for more therapy individualization and consequently of further research in this field.

## Figures and Tables

**Table 1:** Patient demographic and clinical data.

Patient ID	Gender	Age (a)	Weight (kg)	Administered Activity (GBq)	Tumour Type	TNM Staging
IP1	f	44.7	85	3.55	papillary	pT1a, pNx
IP2	f	65.3	72	3.51	papillary (follicular variant)	pT1b, Nx
IP3	f	24.9	67	3.55	papillary	pT2m, N1a (3/6)
IP4 *	f	28.6	78	3.57	papillary	pT1b, pNx
IP5	f	55.2	46	3.43	papillary	pT1b, pNx
IP6 *	f	21.0	115	3.52	follicular (encapsulated angio-invasive)	pT1b, Nx
IP7	f	54.7	70	3.72	papillary	pT2m, pNx
IP8	f	48.8	60	3.65	papillary	T2, Nx
IP9 *	f	45.1	108	3.50	papillary	pT2(m), pN0 (0/1)
IP10	f	44.6	77	3.56	papillary	pT2, N0 (0/10)
IP11	m	59.9	80	3.58	papillary	pT1b, pN0 (0/12)
IP12	f	26.2	85	3.56	papillary	pT1b, pN0 (0/3)
IP13	m	36.3	96	3.60	papillary	pT2, pNx
IP14	f	38.9	68	3.91	papillary	pT1b, pN1b (3/22)
IP15 * ^†^	m	54.7	94	3.52	papillary	pT3a, pNx
IP16	f	63.8	85	3.60	follicular	pT2, pN0 (0/6)
IP17	m	19.8	82	3.66	papillary	pT1b, pN0
IP18	f	59.2	96	3.60	papillary	pT2, pN1b (2/33)

* These patients received a second radioiodine therapy. ^†^ This patient received rhTSH before treatment.

**Table 2:** Median absorbed dose to the blood and absorbed dose rate and median of the average number of RIF per cell at the nominal time points investigated.

Nominal Time Point	Median Absorbed Dose to the Blood (mGy)	Median Absorbed Dose Rate (mGy h^−1^)	Median of the Average Number of RIF per Cell	Number of Patients with RIF-Data
0 (baseline)	0	0	0	18
1 h	14 (12–19)	15.5 (8.1–20.0)	0.37 (0.16–0.74)	18
2 h	29 (24–37)	14.7 (12.3–18.3)	0.61 (0.26–0.88)	17
3 h	43 (37–55)	13.9 (10.2–16.7)	0.48 (0.27–0.83)	18
4 h	57 (48–71)	12.8 (8.7–15.3)	0.66 (0.33–0.99)	18
24 h	200 (144–239)	3.5 (2.5–5.4)	0.41 (−0.07–0.81)	18
48 h	249 (192–323)	1.1 (0.5–2.2)	0.26 (−0.12–0.60)	18
96 h	278 (203–385)	0.2 (0.0–1.9)	0.15 (−0.09–0.34)	12
168 h	287 (205–419)	0.1 (0.0–1.1)	0.05 (−0.21–0.38)	17
∞	299 (205–605)	--	--	--

**Table 3:** Modelling fit parameters: separate fits of all patients.

Patient ID	*c* (mGy^−1^)	*α*	*k*_1_ (h^−1^)	*k*_2_ (h^−1^)	*N*_0_	*r* ^2^	Number of Data Points	Group	Ex Vivo *R* (h^−1^) [17]
IP1	0.012 ± 0.001	0.931 ± 0.036	0.210 ± 0.038	0.018 ± 0.011	0.004 ± 0.015	0.99	9	1	0.19 ± 0.04
IP2	0.023 ± 0.008	0.942 ± 0.031	0.389 ± 0.231	0.006 ± 0.007	−0.005 ± 0.080	0.91	9	1	0.19 ± 0.01
IP3	0.023 ± 0.005	0.945 ± 0.020	0.324 ± 0.156	0.006 ± 0.005	0.015 ± 0.060	0.96	9	1	0.23 ± 0.15
IP4	0.071 ± 0.014	0.980 ± 0.006	1.330 ± 0.302	0.017 ± 0.008	0.003 ± 0.030	0.99	9	2	0.12 ± 0.03
IP5	0.028 ± 0.009	0.986 ± 0.017	0.499 ± 0.226	0.000 ± 0.011	−0.020 ± 0.090	0.94	9	1	0.10 ± 0.08
IP6	0.109 ± 0.125	0.954 ± 0.050	3.028 ± 3.811	0.026 ± 0.010	−0.009 ± 0.044	0.98	8	2	0.43 ± 0.32
IP7	0.024 ± 0.017	0.943 ± 0.057	0.554 ± 0.596	0.016 ± 0.028	−0.103 ± 0.148	0.81	9	1	0.24 ± 0.13
IP9	0.047 ± 0.056	0.950 ± 0.091	1.479 ± 2.259	0.040 ± 0.097	−0.029 ± 0.098	0.84	8	2	0.16 ± 0.03
IP13	0.044 ± 0.031	0.959 ± 0.027	1.262 ± 0.993	0.006 ± 0.005	−0.004 ± 0.085	0.87	9	2	0.35 ± 0.04
IP14	0.047 ± 0.031	0.924 ± 0.056	1.140 ± 1.004	0.031 ± 0.024	−0.042 ± 0.091	0.92	9	2	0.27 ± 0.08
IP15	0.019 ± 0.006	0.979 ± 0.061	0.193 ± 0.102	0.000 ± 0.030	0.018 ± 0.083	0.85	9	1	0.19 ± 0.08
IP16	0.021 ± 0.005	0.958 ± 0.011	0.451 ± 0.178	0.000 ± 0.003	−0.003 ± 0.051	0.96	9	1	0.18 ± 0.03
IP17	0.051 ± 0.031	0.935 ± 0.045	1.393 ± 1.117	0.044 ± 0.030	−0.049 ± 0.050	0.95	9	2	0.18 ± 0.11
IP18	0.020 ± 0.006	1.000 ± 0.020	0.217 ± 0.077	not applicable	−0.033 ± 0.109	0.90	9	1	0.20 ± 0.11

**Table 4:** Modelling fit parameters: comparison between Group 1 and Group 2.

Group	*c* (mGy^−1^)	*α*	*k*_1_ (h^−1^)	*k*_2_ (h^−1^)	*N_0_*	*r* ^2^	Number of Data Points
1	0.022 ± 0.004	0.954 ± 0.015	0.362 ± 0.109	0.006 ± 0.005	0.000 ± 0.038	0.73	72
2	0.065 ± 0.024	0.962 ± 0.016	1.546 ± 0.669	0.027 ± 0.012	0.001 ± 0.035	0.81	52

## Data Availability

The datasets generated and analyzed in the course of the current study are available from the corresponding author on reasonable request. Part of the results have been published as an interim report/deliverable during the MEDIRAD project [18].

## Supplementary Materials

The following supporting information can be downloaded at: https://www.mdpi.com/article/10.3390/ijms25105493/s1.
