# Supplementary material for: Modelling the In Vivo and Ex Vivo DNA Damage Response after Internal Irradiation of Blood from Patients with Thyroid Cancer
Source: Int J Mol Sci. 2024 May 17;25(10):5493. doi: 10.3390/ijms25105493 (PMC11122196; doi:10.3390/ijms25105493)
Supplement: Supplementary file 1 [file ijms-25-05493-s001.zip › ijms-2940591-supplementary.pdf]

Modelling the In Vivo and Ex Vivo DNA Damage Response after Internal Irradiation of Blood from Patients with Thyroid Cancer

Supplemental Data

Sarah Schumann[1], Harry Scherthan[2], Philipp E. Hartrampf[1], Lukas Göring[1], Andreas K. Buck[1], Matthias Port[2], Michael Lassmann[1] and Uta Eberlein[1]

1) Department of Nuclear Medicine, University Hospital Würzburg, 97080 Würzburg, Germany

2) Bundeswehr Institute of Radiobiology affiliated to the University of Ulm, 80937 Munich, Germany

Patient Inclusion and Exclusion Criteria

1. **Inclusion criteria**
    - 1.1. Able and willing to give signed informed consent
    - 1.2. Age ≥ 18 years
    - 1.3. Total thyroidectomy performed within a maximum of 6-8 weeks before radioiodine treatment
    - 1.4. Histological evidence for differentiated thyroid carcinoma (DTC) (papillary, follicular variants) (histological variants of aggressive disease such as tall cell, columnar, hobnail, diffuse sclerosing variants will be excluded)
    - 1.5. Tumor staging: T1b, T2, T3a, any N (in accordance with the 8th ed. of the AJCC/UICC TNM staging, 2017
    - 1.6. No distant metastases (patients who will be diagnosed with distant metastases on the post-therapy scan will be replaced)
    - 1.7. Clinical indication for I-131 therapy as determined by the attending physician based on the available clinical information
    - 1.8. WHO score, ECOG score 0-2 (autonomous, self-caring)
    - 1.9. No history of prior therapeutic radiation / radionuclide exposure
    - 1.10. No history of chemotherapy
    - 1.11. No medication known to interfere with iodine kinetics within the last 12 months
    - 1.12. No amiodarone exposure in the last 24 months
    - 1.13. No exposure to iodinated contrast agent in the last six months
    - 1.14. No hemodialysis

2. **Exclusion criteria:**
    - 2.1. Pregnancy (to be excluded by serum beta-HCG measurement in women of child-bearing age)
    - 2.2. Lactation within the last 6 months
    - 2.3. Participation in another clinical study within the last 6 months
    - 2.4. current uncontrolled severe disease
    - 2.5. History of prior malignancy with the exception of low-grade skin cancer
    - 2.6. Inability to tolerate the necessary measurements for the study
    - 2.7. Diseases of the hematopoetic system
    - 2.8. Diagnostic exposure to ionizing radiation within one week of planned exposure to I-131

Cluster plot

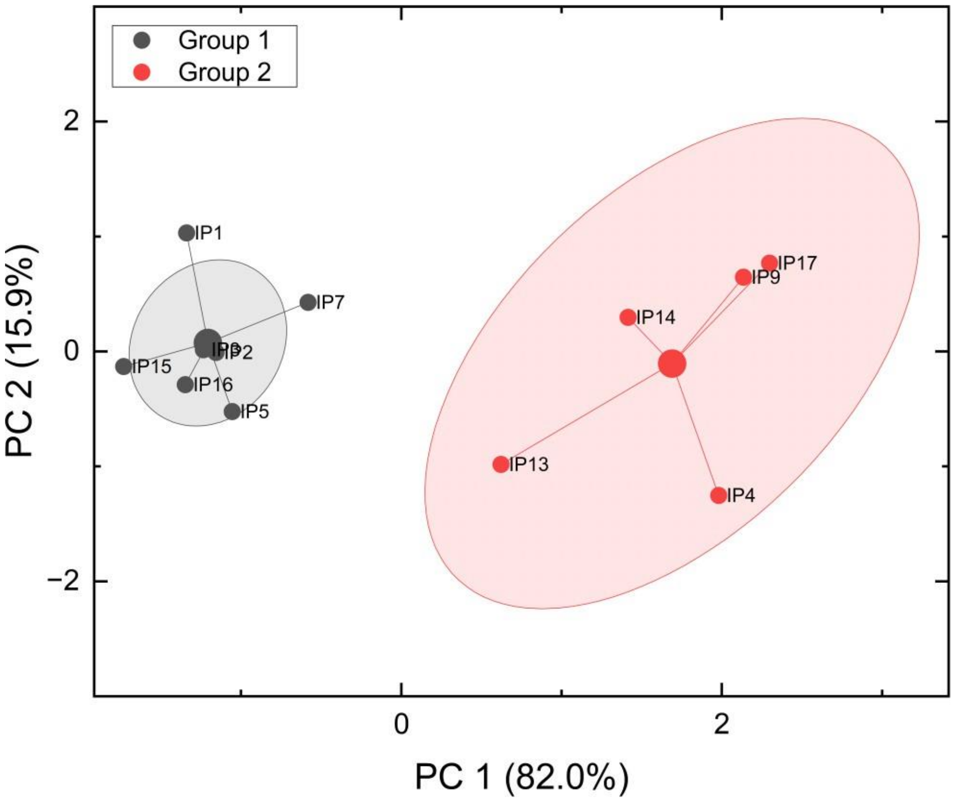

Supplemental Figure S1: Cluster plot of the k means cluster analysis for k_1, k_2, and c for Groups 1 and 2. The shaded areas denote the 95% confidence area.

References

1 Schumann, S. *et al.* DNA damage and repair in peripheral blood mononuclear cells after internal ex vivo irradiation of patient blood with ^{131}I. *Eur J Nucl Med Mol Imaging* **49**, 1447-1455 (2022). https://doi.org:10.1007/s00259-021-05605-8
